# The floodplain inundation history of the Murray-Darling Basin through two-monthly maximum water depth maps

**DOI:** 10.1038/s41597-023-02559-4

**Published:** 2023-09-23

**Authors:** David J. Penton, Jin Teng, Catherine Ticehurst, Steve Marvanek, Andrew Freebairn, Cherry Mateo, Jai Vaze, Ang Yang, Fathaha Khanam, Ashmita Sengupta, Carmel Pollino

**Affiliations:** 1 Commonwealth Scientific and Industrial Research Organisation, Adelaide, Australia; 2 Murray-Darling Basin Authority, Canberra, Australia

**Keywords:** Hydrology, Environmental sciences, Natural hazards

## Abstract

With growing concerns over water management in rivers worldwide, researchers are seeking innovative solutions to monitor and understand changing flood patterns. In a noteworthy advancement, stakeholders interested in the changing flood patterns of the Murray Darling Basin (MDB) in Australia, covering an area of 1 million km^2^, can now access a consistent timeseries of water depth maps for the entire basin. The dataset covers the period from 1988 to 2022 at two-monthly timestep and was developed using remotely sensed imagery and a flood depth estimation model at a spatial resolution of ≈30 m, providing a comprehensive picture of maximum observed inundation depth across the MDB. Validation against 13 hydrodynamic model outputs for different parts of the MDB yielded a mean absolute error of 0.49 m, demonstrating reasonable accuracy and reliability of the dataset. The resulting dataset is best suited to system-wide analysis but might also be useful for those interested in the history of flooding at specific locations in the system. We provide the dataset, visualization tools, and examples to support ongoing research.

## Background & Summary

Accurate and detailed flood depth data plays a vital role in understanding flood processes, assessing flood hazards, and managing ecosystem services effectively. However, acquiring such data through extensive hydrodynamic modelling can be financially burdensome. For instance, the National Flood Insurance Program of the United States of America has invested approximately $11 billion USD over five decades to develop Flood Insurance Risk Maps for a third of its river systems^1^. In response to the global challenge of obtaining flood information, researchers have developed methods involving global datasets^2^, simplified solutions to hydrodynamic equations^3^ and hybrid remote-sensed approaches^4^, which are often distributed through information systems such as the Global Flood Inundation Map Repository and local hubs such as the US Flood Inundation Map Repository^5^. This work is a notable case study in this endeavour.

The Murray-Darling Basin (MDB) is a vast geographical region in the south-eastern part of Australia that encompasses the drainage basins of the Murray River and the Darling River, which are respectively Australia’s longest and second-longest rivers^6^. In total, the MDB covers approximately 14% of Australia’s land area. It provides around 40% of Australia’s total value of agricultural production each year^7^ and is of global ecological significance for its Ramsar-listed wetlands. The MDB has been the site of significant political debate between agricultural, social, and ecological interests^8^. At its heart, this debate is motivated partly by questions that remain over the volume of water that irrigators should divert for consumption and the volume of water that should be provided to maintain healthy rivers and functioning floodplain ecosystems^9^. Part of the debate is value-based – some people value ecosystems more or less than other people. However, there are also fundamental gaps in our understanding of how floodplain ecosystems function and how they change through cycles of floods and droughts.

What we do know is that floodplain ecosystems of the MDB rely on fluvial overbank flooding. Fluvial overbank flooding refers to the flooding that occurs when a river or stream overflows its banks and inundates adjacent land areas. This type of flooding is common in low-lying areas and is important to maintain ecological processes. For example, flood frequency, timing, depth and duration are known to drive ecosystem processes such as *Eucalyptus Camaldulensis* (River Red Gum) growth, viability of *Muehlenbeckia florulenta* (Lignum) seeds and bird breeding events^10,11^. Many of these findings have been derived from greenhouse experiments (i.e. measuring the response to controlled conditions such as flooding seedlings for 40 days^12^) and/or local field studies (e.g. testing the viability of seeds in Morgan, South Australia^13^). Extrapolating these findings across the million square kilometres of the MDB is difficult or problematic because of a lack of consistent and systematic information on floodplain hydrology, among other things.

In recent years, techniques for generating consistent and systematic information for fluvial systems have advanced. For example, mapping of flood extent using optical remote sensing techniques have improved^14^ and become widely accessible (e.g. Geoscience Australia’s Water Observations^15^). The next step should be to provide the depth of floodwater systematically because the depth of floodwater is as important as extent when establishing ecological relationships (as well as flood risks, impacts and recovery efforts). However, the depth of water during flood events is harder to obtain, or unavailable, especially when users require continuous data over large spatial domains at a fine spatial resolution (<50 m).

Traditionally, modellers have used two-dimensional hydrodynamic models to calculate floodwater depth, but the models require detailed flow, morphology, and roughness information, and the computational costs become prohibitively high for regions as large as the MDB. As such, there is currently no flood depth dataset available for the basin. Teng *et al*.^16^ conducted a comprehensive assessment of floodwater depth estimation models in semi-arid regions and found that the Floodwater Depth Estimation Tool (FwDET), first developed by Cohen *et al*.^17^ was the most suitable approach for this region.

We estimated flood water depth with FwDET and estimated the product accuracy using hydrodynamic models for a selection of floodplains. The resulting product provides spatial layers that represent maximum observed surface water extent and water depth within each two-month period across the MDB at a spatial resolution of ≈30 m from January 1988 to December 2022.

**Fig. 1:**
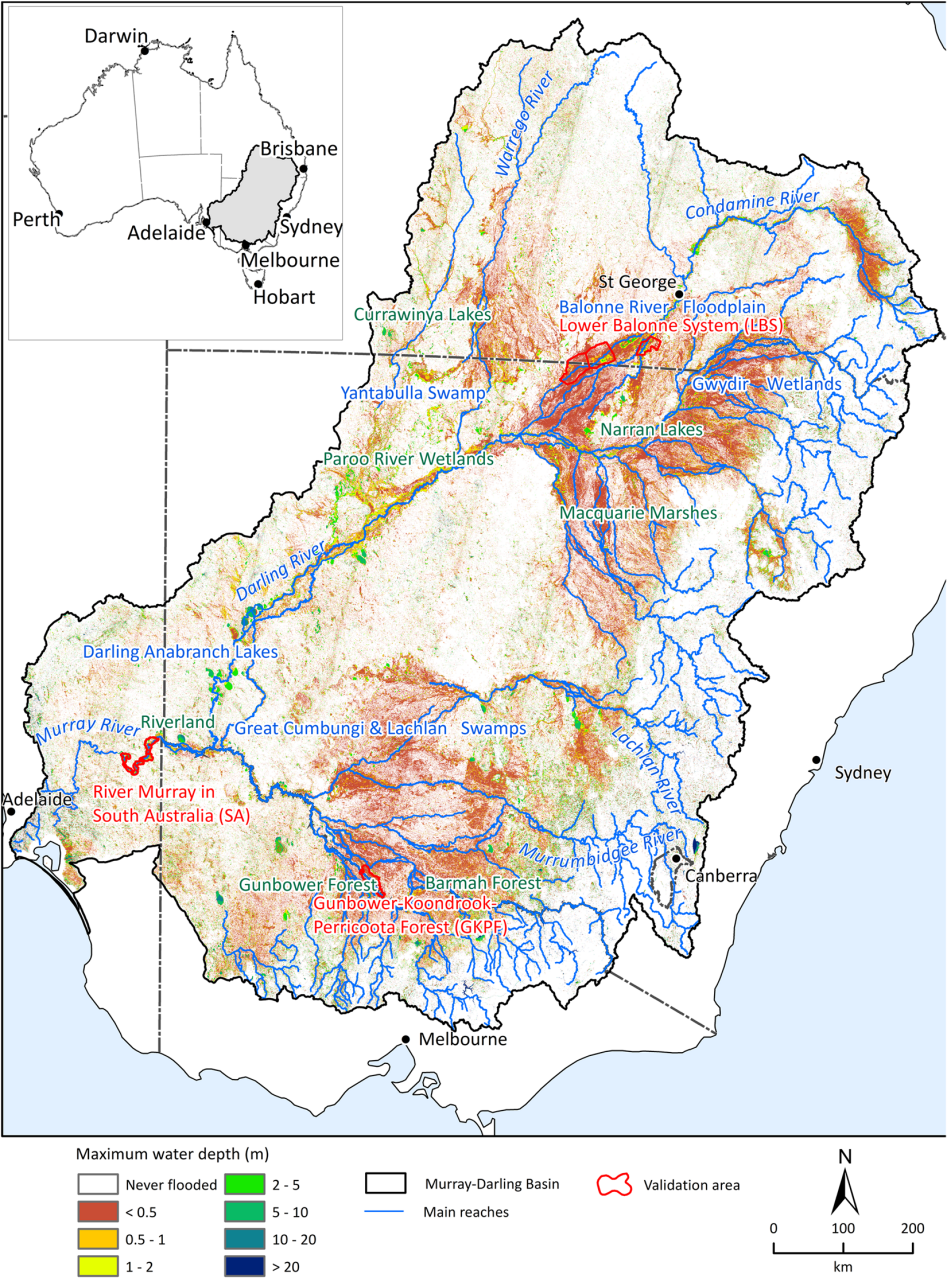
Maximum floodwater depth. The maximum floodwater depth for the Murray Darling Basin calculated from the two-monthly floodwater depth dataset.

The resulting dataset can be used to investigate links between flooding and ecological functions. It is best suited to analysing the whole MDB over significant periods of time. For example, the dataset is suited to studies interested in the physical and biological connectivity of the floodplain and how that connectivity has changed over time. It is also suited to developing empirical relationships between flooding and ecosystems processes. It might also be useful for those interested in the history of flooding at specific locations in the river system, although it would need to be cross-checked with local data to confirm its accuracy. An example of maximum floodwater depth calculated over the 35 years is shown in Fig. 1. Linear features running in an approximate north-south direction are visible in Fig. 1. This occurs along the swath edge of the Landsat 7 images (during its later years of operation) and are noise where the pixels are erroneously classified as water.

Other applications include development of coarse scale hydrodynamic models and ecosystem services valuations. Coarse scale hydrodynamic models provide insights into flood hazard and exposure^18^. When building hydrodynamic models, floodwater extents and depths are used for calibration and validation. Given the size of the MDB, a major factor limiting the generation of hydrodynamic models is the time consumed collating regionwide floodwater extents and depths, which this product provides. Similarly, this product aims to contribute valuable insights for ecosystem services valuations. Accurate estimations of water availability are crucial for assessing floodplain productivity and its various aspects, including floodplain grazing, tourism, mental health outcomes and First Nation’s cultural economies. These valuations help to understand the broader impacts of floodplain management and inform decision-making processes. By linking floodplain inundation to social and economic outcomes, typically at the local government level, we gain a comprehensive understanding of the benefits derived from floodplain resources and support ongoing management strategies.

The findings presented in this study have significant broader impacts for communities, flood managers, decision/policy makers, and stakeholders in the MDB. The dataset of consistent timeseries water depths provides valuable insights into changing flood patterns and their impact on MDB’s floodplain ecosystems. Flood managers can improve flood management strategies, while decision/policy makers can develop effective flood mitigation plans. Communities can gain knowledge on flood risks to direct resilience efforts. Scientific organisations and environmental conservation initiatives, can benefit from assessing the impacts of changing floods on biodiversity. This study facilitates evidence-based decision-making and collaboration, supporting ongoing research in the MDB.

## Methods

**Fig. 2:**
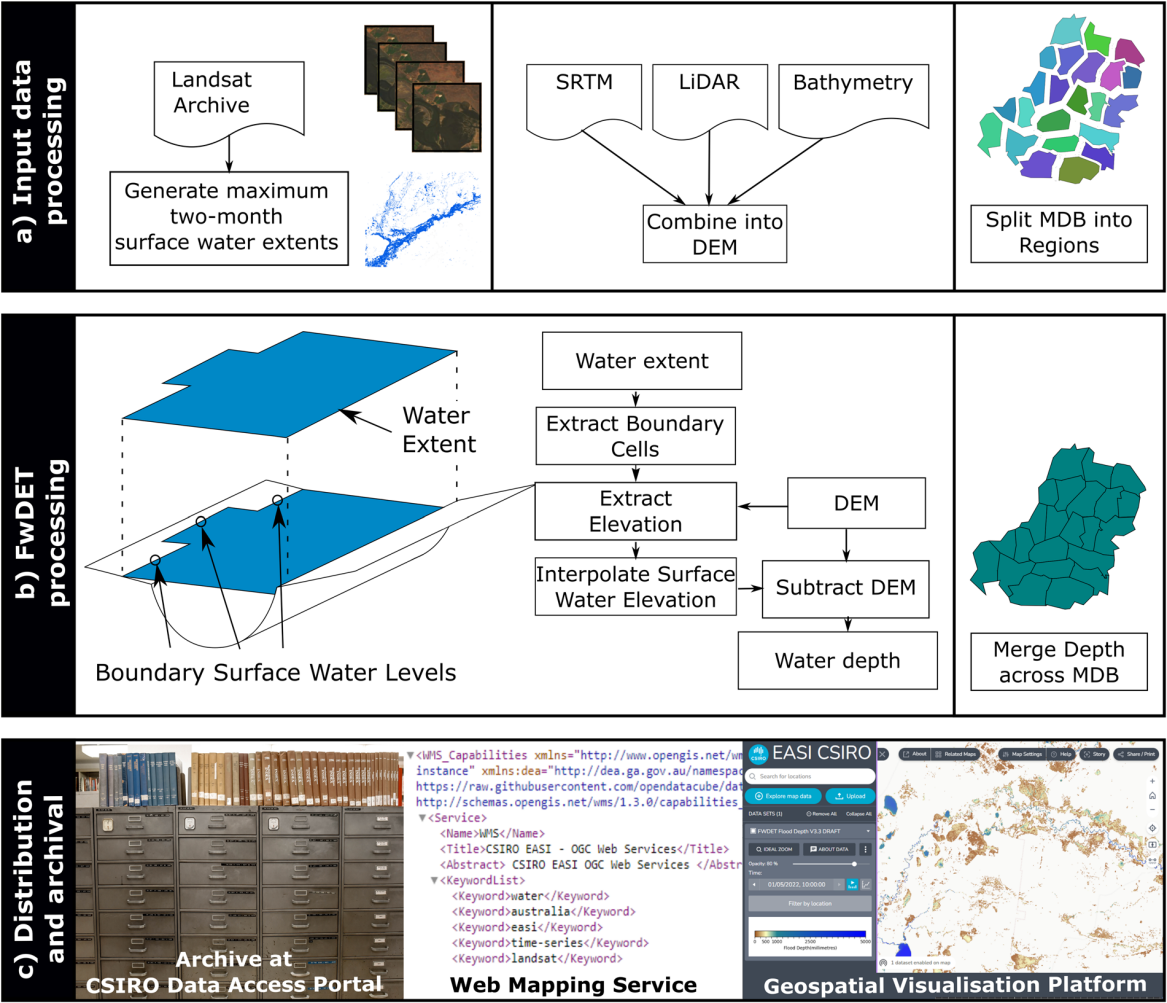
Steps involved in building and distributing the flood water depth product for the Murray Darling Basin. The three panes show steps of the water depth product development. (**a**) Input data processing involves acquisition of two products: two-monthly maximum water surface extent (from Landsat)^19^ and a high-resolution Digital Elevation Model (combined from data sources)^20^, which we split into 23 regions for processing. (**b**) Floodwater Depth Estimation Tool (FwDET) algorithm v2^32^ was used to identify the surface water elevation at the boundary (perimeter) of inundated areas. The perimeter water surface levels (elevations) were interpolated across inundated areas to provide continuous surface water levels. The depth was calculated by subtracting the Digital Elevation Model from the surface water levels and merging (recombining) across the Murray Darling Basin. (**c**) The resulting water depth rasters were archived in CSIRO’s Data Access Portal, and were also distributed through web services for machine access (i.e. Web Mapping Service) and presented through a geospatial visualisation platform for point-and-click visualisation of water depth across the floodplains of the Murray Darling Basin.

Figure 2 shows the workflow that generated flood depth products using the FwDET algorithm. Details of the FwDET inputs, implementation, archiving, and visualisation are in the sections below.

### Data inputs

Inputs to the FwDET algorithm were a spatial time series of two-monthly maximum surface water extents^19^ and a digital elevation model (DEM) with bathymetry that covers the entire region^20^ (left and middle in Fig. 2a). The two-monthly maximum surface water extents were based on classification of Digital Earth Australia’s Landsat Surface Reflectance collection^21^. The collection provided satellite images of high quality and resolution (30 m × 30 m) from 1988 until the present. The Landsat Surface Reflectance product contained surface reflectance values for 7 spectral bands in the optical range (from the Landsat sensor aboard each of the Landsat missions). Sensors in the optical range could not see the ground surface through cloud. We found that the highest temporal frequency that would ensure there were cloud-free observations for almost all pixels was every two months. Hence, we selected the maximum observed extent for each two-monthly period.

There are many water indices that quantify the likelihood of surface water given the spectral properties of Landsat cells including the modified Normalised Difference Water Index^22^ and Fisher’s Water Index^23^. These index values can then be compared to thresholds to classify Landsat cells as wet or dry. We used a multi-index method^14^ for water classification that selected appropriate water indices and thresholds for different land types (e.g. river, floodplain, wetland) using a decision tree. The multi-index method had a water pixel accuracy of 90.5% and a dry-pixel accuracy of 94.8% (with an overall Kappa statistic of 0.88) when validated against 440 cloud-free plots of 300 m × 300 m size in the MDB. This has ranked it the best of 8 surveyed indices and thresholds for water classification in the MDB.

The resolution and accuracy of the DEM played a major role in the reliability of the estimated water depth. The selected DEM^20^ had a resolution of 2.698 × 10^−4^ degrees in WGS84 projection (approximately 30 m). The DEM^19^ was produced by merging and blending high resolution LiDAR DEMs with a hydrologically enforced Shuttle Radar Topography Mission (SRTM) DEM^24^ where LiDAR was not available. The high-resolution LiDAR was aggregated to the common resolution using the mean of contributing cells. Off the shelf global low resolution DEMs such as SRTM, FABDEM (Forest And Buildings removed Copernicus DEM)^25^ and MERIT (Multi-Error-Removed Improved-Terrain)^26^ cannot represent large channels due to their coarse pixel resolution and post-processing is required to correction for errors along rivers. The SRTM product was specifically post-processed for the Australian region and hydrologically enforced. Hydrological enforcement used existing mapped drainage lines to modify the SRTM to properly represent flow paths in the landscape. The accuracy of the DEM was important as water depth was derived from the difference between water level and DEM derived ground level (panel b in Fig. 2).

The merging process was undertaken in a similar manner to other products for the Australian landscape^27^. The merging process blended DEMs so that the highest resolution/accuracy was achieved wherever possible and the abrupt changes at the border of two datasets attenuated within a 2 km buffer. The South Australian LiDAR DEM had accurate river bathymetry from Sonar acquisitions. Where bathymetry was missing in permanently inundated channels and wetlands, the channel depth was estimated using gauged water level from the Bureau of Meteorology’s Water Data Online. The channel depth was then added to the DEM to take into consideration the areas that were under water when the DEM was collected^20^. Finally, the classified water pixels were assigned to the nearest DEM cell so that everything aligned.

The vertical accuracy of the LiDAR, which covered much of the floodplain areas, depended on the point density of the LiDAR acquisition, the accuracy of the ground control points and the density of ground cover points. There were around 10 sources of LiDAR collected over the last 20 years and available through Geoscience Australia or the Murray Darling Basin Authority. The accuracy varied between products; however, when compared to survey points (accurate to 0.1 m), one LiDAR product had a root mean squared error (RMSE) of 0.206 m for hard bare surfaces^28^. For regions away from the floodplain, the accuracy from SRTM was likely to be in the range of RMSE from 5 to 15 m^29^ with some improvements due to the hydrological enforcement and smoothening to remove vegetation features and noise^30^.

### Application of the Floodwater Depth Estimation Tool

The input datasets for the MDB (water extents and DEM) were broken into 23 regions to reduce processing memory requirements. The regions were defined along catchment boundaries to minimise splitting flooded areas that would create discontinuity. Specifically, the 19 Murray-Darling Basin Sustainable Yields (MDBSY) project reporting region^31^ boundaries were used as a starting point as they were already derived from hydrological basins. The maximum flood extent was overlaid over the MDBSY regions, which were either combined, subdivided and/or reshaped accordingly to derive the 23 regions used in the further processing. The rezoning was guided by the following principles:

1. Where possible, the zones were split at points that maintained the contiguous areas of the maximum flood extent.
2. Where splitting of maximum flood extent was unavoidable (e.g. the Murray trench), the split was implemented where the flood extent was narrowest.
3. Zone shape and size were optimised to avoid large areas of no-data occurring in the rectangular extent of each zone.

The resulting 23 analysis zones are shown in the top right panel of Fig. 2 (and included in with the final product as auxiliary data).

The FwDET v2.0 algorithm^32^ (hereafter referred to as FwDET) was then applied to generate water depths as shown in Fig. 2b. FwDET calculates water depth by first extracting boundary elevation of the flood extent from a DEM, interpolating water surface elevation using the boundary elevation, then subtracting the ground elevation from the interpolated surface elevation. The interpolation method used in this version was the Thin Plate Spline, which provided a smoother surface and overcome some of the known caveats of linear interpolation used in the previous version of FwDET^16^. Input data borders and the boundaries of areas without data (fully cloud covered through the two-month period) were not included as points in the interpolation.

FwDET and the associated workflow were written in Python and made suitable for parallel computing for a large spatial domain at a high spatial resolution.

### Data archiving and visualization

The results were archived in CSIRO’s Data Access Portal and are available directly from the archive, through web services or via an online visualisation tool. The CSIRO’s Data Access Portal has been certified as a trusted data repository by CoreTrustSeal.

The dataset was then prepared for online access, visualisation and presentation. First the dataset was reprojected into a 25 m grid in Web Mercator Projection and housed on Amazon Web Services S3 storage. Then the dataset was indexed using the Open Data Cube libraries^33^ on the Earth Analytics Science and Innovation (EASI) platform^34^. The Python datacube-ows package (https://github.com/opendatacube/datacube-ows) was used to provide an Open Geospatial Corporation Web Map Service^35^ to the indexed dataset^36^. Finally, water depth layers were prepared for the Terria map library and are available online for visualisation.

## Data Records

The water depths are hosted on the CSIRO Data Access Portal at: https://data.csiro.au/collection/csiro:50243^37^. There are a total of 213 files summing to around 23.19 GB of data. Inside the MDB_Water_Depth_v3.6_4326 folder are files for each spatial map of maximum water extent and depth for the Murray-Darling Basin for a two-month period starting in January 1988 and finishing in December 2022. The January-February image for 1988 has the date label ‘1988-01’, the March-April image for 1988 has the date label ‘1988-03’, and so on. The files are stored in Cloud Optimised GeoTiff (COG) format in WGS84 – (EPSG:4326) projection. The COG files represent depth in millimetres as a 16-bit unsigned integer (uint16). The maximum depth value is limited to 65534 mm. The minimum water depth is 1 mm. The value 0 represents dry land and the value 65535 is reserved for no-data. No-data values occur when there were no cloud-free days observed in the input flood extent product.

Inside the Auxiliary folder are supporting files: 1) MDB_Sub_Div_23zone_1sec.tif and 2) MDB_permanent_water_correction.tif. The first geotiff file is the 23 sub-divisions of the MDB used during processing with each sub-division numbered and encoded as an 8-bit integer. The second geotiff file is the bathymetric correction values (in meters) that were applied to the DEM encoded as 32-bit floating point values. We have provided these to complete the provenance trail.

## Technical Validation

Validation of the dataset in the MDB was established by benchmarking the FwDET-derived depth outputs to hydrodynamic models and reviewing the dataset with experts to identify errors.

### Selection of hydrodynamic models for benchmarking

**Fig. 3:**
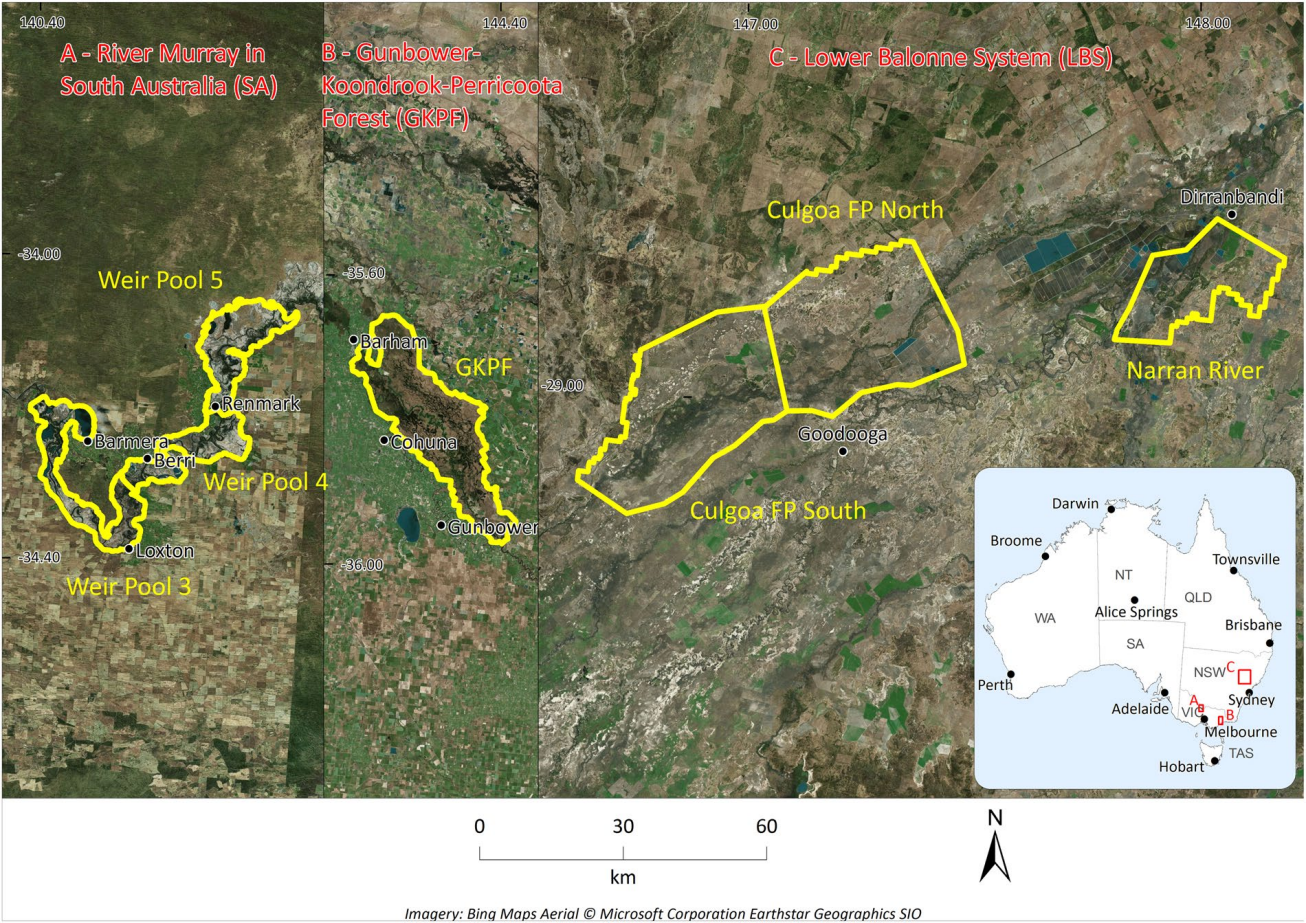
Location of validation sites. These include three reaches of the River Murray in South Australia (**A**), one reach of River Murray in Victoria (**B**), and three reaches of the Balonne River (**C**), where the water depth data were compared to benchmarking hydrodynamic modelling results.

After we had conducted a thorough review of 13 hydrodynamic models for the MDB^16^, we carefully selected three representative hydrodynamic models^38–40^ that covered different parts of the MDB, as illustrated in Fig. 3. The first model was chosen to represent the floodplains of the Lower Balonne System (LBS) in Queensland and New South Wales, while the second model represented the floodplains of the Gunbower-Koondrook-Perricoota Forest (GKPF) in New South Wales and Victoria. The third model encompassed the floodplains downstream of Lock 6 and upstream of Lock 3 on the River Murray in South Australia (SA).

The selection process was guided by several factors, including input data quality, validation against gauge height measurements, peer review of the model setup, and availability to the project team. These models were also specifically chosen to be representative of different environments across the Murray Darling Basin. To ensure robust and reliable data sources, the assessment of input data quality involved evaluating the accuracy, resolution, and coverage of the data utilised by each hydrodynamic model. The selected models utilised high-resolution Digital Elevation Models (DEMs) based on LiDAR with high-quality bathymetry obtained from sonar, LiDAR, or manual cross sections.

Furthermore, the validation process involved comparing the model outputs to observed data from gauges to assess their performance in replicating real-world hydrological conditions. For instance, the Balonne River median model exhibited an R^2^ ranging from 0.6 to 0.8 when compared to time series observations of water level in four streamflow gauges within the floodplain during the occurrence of four floods.

The peer review of model setup played a crucial role in establishing confidence in the technical soundness and appropriateness of each model’s configuration. This was evident in the South Australian model development process. The South Australian model was revised several times to support its use in designing physical constraints for improved delivery of environmental water.

While the selected models offer valuable insights, the inclusion of additional models would enhance the overall reliability and provide further understanding of the complex hydrodynamics within the basin. Future studies might consider incorporating a broader ensemble of models to advance our understanding of the MDB’s hydrodynamics and to account for the inherent uncertainties in modelling.

### Description of flood events used for validation

Floods of the Lower Balonne region have been caused by large rainfall events across the upstream catchments. The region has been susceptible to flooding during Australia’s seasonal wet monsoon, which has typically lasted from December to March. This monsoonal flooding has been amplified by the effect of La Niña climate drivers. The LBS model represented the floods that began in January 1996, January 2011 and January 2012. The January 1996 flood event was influenced by tropical Cyclone Barry which developed in the Gulf of Carpentaria in early January and spread from the Gulf to the south-east corner of Queensland producing widespread rainfall and flooding (17 major rivers recorded flooding)^41^. During the 2010–2011 La Niña event, one of the strongest on record, the region experienced heightened rainfall in this catchment and across most of Queensland with an average 200 mm falling across the entire state in December^42^. Similarly, the January 2012 flood resulted from a persistent trough and the development of a monsoon low, which caused prolonged and intense rainfall (event totals of 300 to 400 mm)^43^. This resulted in flood dynamics in the Lower Balonne floodplain that were intricate because multiple upstream river systems contributed to the overbank flooding. The peak heights of the three events at St George gauge (422201) were 10.6 m on 1996-01-20, 12.8 m on 2011-01-08 and 13.5 m on 2012-02-07 with estimated discharges of 1872 m^3^s^−1^, 2964 m^3^s^−1^, and 3963 m^3^s^−1^. The floodwaters remained at major flood levels (overbank) for a number of weeks.

The SA and GKPF models simulated flooding during the November 2016 period. Earlier, in September, the Murray-Darling Basin experienced its wettest September on record with average aerial rainfall of 119 mm, which was 249% above the long-term average^44^. This continued through to November and marked a shift from drought to flood. The heavy rainfall was widespread and affected both the upper catchments along the eastern mountain range of the MDB (i.e. Great Dividing Range) and the broader Basin area. It was attributed to strong cold fronts in the south and trough systems connected to tropical moisture from above-average sea-surface temperatures in the Indian Ocean and northern/eastern Australia^45^. The calculated discharge at the South Australian border (A4261001) was 1095 m^3^s^−1^ on 2016-11-30. The discharge from upstream rainfall events resulted in a continued period of high flows - flows over 1000 m^3^s^−1^ were recorded for about 3 weeks.

### Comparison to hydrodynamic model outputs

From the three hydrodynamic models, we extracted surface water elevation outputs from calibration scenarios for seven regions (see Fig. 3). The benchmark results for the comparison were calibration runs of dynamic (unsteady) hydrodynamic model scenarios. While the hydrodynamic model outputs were not observations, we expected the vertical accuracy of the hydrodynamic model outputs would be superior to the vertical accuracy of FwDET. Following the same technique as Teng *et al*.^16^, the two-monthly maximum depth outputs were translated into surface water elevation (by adding the DEM) and compared at 5 m spatial resolution for each flood event. With the combination of flood events (up to three events per model) and regions, there were 13 model outputs that were part of the comparison. Finally, the estimated water depth was evaluated against the hydrodynamic model output using the RMSE, mean absolute error (MAE) and bias (benchmark minus predicted). These evaluation methods were consistent with previous work^16^ and methods for evaluating elevation errors in DEMs^46^.

**Fig. 4:**
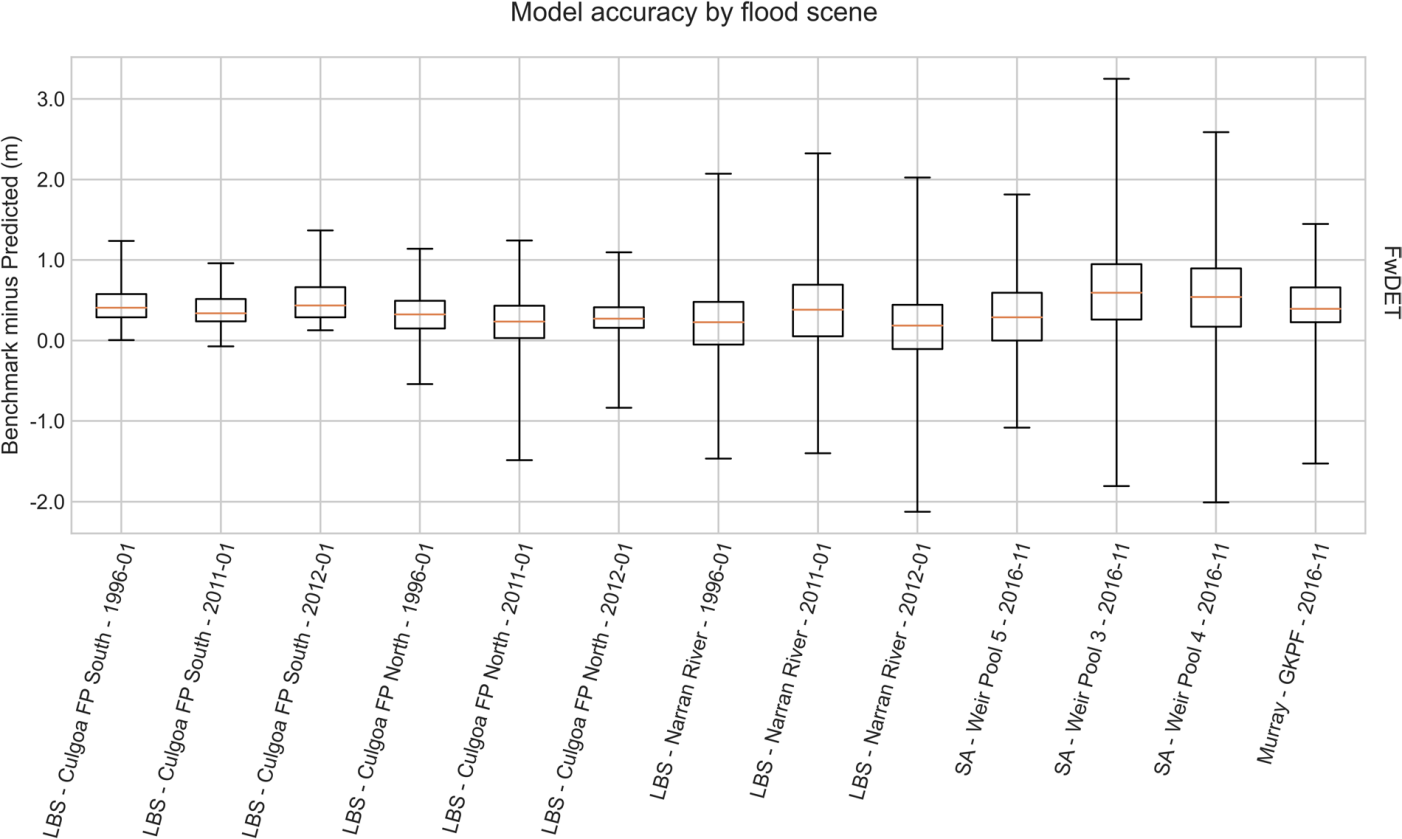
Floodwater depth accuracy for each flood scene. The accuracy is expressed as benchmark minus prediction. The positive values represent underestimation of depth and negative values overestimation. Acronyms on axis labels are Lower Balonne System (LBS), Floodplain (FP), Gunbower Koondrook Perricoota Forest (GKPF) and South Australia (SA).

Figure 4 shows the bias (benchmark minus predicted) of the 13 model outputs. In general, FwDET underpredicted the depth of flooding with an average underprediction of 0.32 m and an inter-quartile range (IQR) of 0.23 m–0.39 m. The IQR demonstrated that 75% of values were within 0.09 m of the mean, that is, the underprediction was consistent statistically. In other words, if the mean error were subtracted from the outputs, 75% of values were within 0.09 m of the benchmark model.

As shown in Table 1, the benchmark versus predicted RMSE ranged from 0.48 m to 1.29 m and MAE ranged from 0.36 m to 0.87 m across the flood events. The median MAE was 0.49 m with an IQR of 0.39 m to 0.54 m (established through statistical bootstrapping). The MAE demonstrated reasonable accuracy and reliability for a dataset of this spatial and temporal coverage. A greater level of accuracy would be required for emergency management or the engineering of flood protection levees (e.g. +/− 0.15 m). However, the level of accuracy demonstrated is sufficient for understanding ecosystem processes, regional planning and decision-making, especially when used to compare scenarios, infer correlations or develop causative hypotheses.

**Table 1:** Error statistics for the Murray Darling Basinwater depth vs hydrodynamic model benchmark.

Model, region, and flood event	Mean Absolute Error (m)	Root Mean Squared Error (m)
Lower Balonne System - Culgoa Floodplain North - 1996-01	0.43	0.53
Lower Balonne System - Culgoa Floodplain North - 2011-01	0.39	0.53
Lower Balonne System - Culgoa Floodplain North - 2012-01	0.36	0.48
Lower Balonne System - Culgoa Floodplain South - 1996-01	0.46	0.55
Lower Balonne System - Culgoa Floodplain South - 2011-01	0.39	0.46
Lower Balonne System - Culgoa Floodplain South - 2012-01	0.49	0.60
Lower Balonne System - Narran River - 1996-01	0.54	0.78
Lower Balonne System - Narran River - 2011-01	0.60	0.82
Lower Balonne System - Narran River - 2012-01	0.57	0.86
Murray – Gunbower Koondrook Perricoota Forest - 2016-11	0.51	0.64
South Australia - Weir Pool 3 - 2016-11	0.87	1.29
South Australia - Weir Pool 4 - 2016-11	0.78	1.22
South Australia - Weir Pool 5 - 2016-11	0.52	0.83

The best distributions to fit the errors were Laplace distribution (with parameters *µ* = 0.34, *b* = 0.42) or Cauchy distribution (with parameters *x*_*0*_ = 0.34, *γ* = 0.22). The methods and results were consistent with recent model intercomparison^16^. The greatest RMSE and MAE were for South Australian Weir Pool 3 and Weir Pool 4. The impact on model performance was due to the steeply incised river channel and limited cloud-free satellite scenes. The steeply incised river channel meant that FwDET had few accurate points along the perimeter of flooded areas to estimate the height of flood waters.

When considering accuracy outside the boundaries of the validation sites, MAE, which provides a measure of the average difference between predicted and observed values, is expected to be similar. However, RMSE which additionally considers the impact of outliers, may vary depending on the number of samples available for evaluation. The bias is likely to be similar, so to improve the accuracy of depth estimates, it is recommended to apply a bias correction by adding 0.32 m to the predictions. This adjustment aims to address any systematic deviations and align the estimates more closely with the true values. Moreover, to assess the level of uncertainty associated with the predictions, it is possible to estimate uncertainty ranges using Laplace or Cauchy probability distributions. These distributions provide a means to quantify the likelihood of different outcomes and can help in understanding the potential variability in the estimated depths.

### Review of outputs

The outputs were reviewed by the project team and external stakeholders to identify limitations and issues. Four issues were identified: accuracy around permanently inundated lakes, water classification in dense forest, persistent cloud cover and misclassified water pixels. In areas with estimated bathymetry, especially around permanently inundated lakes (e.g. Lake Victoria in South Australia), the water depth can be under or overestimated. In the worst case, the depth layer might have depth values at the edge of the lake that are greater than at the centre of the lake (i.e. the error in depth is as large as the depth of the lake). The adjustment values applied to the bathymetry are provided as a separate raster with the outputs (MDB_permanent_water_correction.tif). The adjustment values can be used as a quality mask to identify those regions where these depth estimation errors are likely to occur. Where the adjustment values are greater than zero, we recommend future research to improve the estimation of bathymetry. Future research could involve collecting sonar bathymetry for permanently inundated regions and collecting LiDAR for ephemerally inundated regions when dry. It may also be possible to infer bathymetry with remote sensing using approaches modified from those applied to coastal regions^47^.

In the areas of Koondrook-Perricoota Forest, and some areas around Barmah Forest, the forest density appears to affect the water classification algorithms. Areas with a closed canopy may be classified as dry by optical remote sensing when they are actually inundated. This has the consequence that the depth product underpredicts inundation. The area south of Koondrook-Perricoota Forest – Gunbower Forest does not appear to have the same issue, so it appears the effect is localised to very densely forested areas of the Murray. Future research could investigate if there are alternate algorithms, remotely sensed indices or thresholds that perform better for water classification, or whether higher resolution optical sensors can improve classification accuracy.

Since the product uses optical remote sensing inputs, persistent cloud cover affects the flood extents used to calculate flood depth. The percentage of cloud cover in a two-monthly image of the MDB ranges from 0.1% up to 38% (which was in the 1990’s when only one Landsat sensor was operating), with an average value of 3%. A two-month period almost always has a cloud-free satellite overpass and major floods in the MDB take months to travel downstream. However, the flood peak is unlikely to be captured perfectly. And, on some occasions, floods may be missed altogether (e.g. the July 1998 flood on the Namoi was not observed). Newer satellites using active sensors in the microwave spectrum (i.e. synthetic aperture radar) can penetrate cloud to provide high resolution imagery of flood waters. In the future, the flood history archive could be augmented with flood extents from these sources.

Misclassified pixels from optical remote sensing inputs propagate with FwDET into erroneous depths. While there has been manual correction of optical classification errors, some misclassifications have remained. When clouds or cloud shadow are classified as water in mountainous and sloped areas, FwDET has generated some abnormally high water depths. Similar artefacts and results have occurred on the edge of satellite swathes and the edge of data impacted by the Landsat 7 EMT + scan line corrector failure.

### Sources of uncertainties and opportunities to extend the methodology

Potential sources of uncertainty in the flood depth products can be attributed to three main factors. First, inaccuracies in the input data, including the DEM and flood extent, led to incorrect depth estimations by the FwDET model. Enhancing the quality and consistency of the DEM, particularly the bathymetry, would significantly improve the accuracy of the results. Prior work compared the performance of LiDAR DEMs to low resolution DEMs across a suite of benchmark models^16^. In that work, the comparative performance across the benchmark suite had a MAE of 0.57 m for LiDAR, MAE of 0.65 m for FABDEM, MAE of 0.71 m for SRTM and MAE of 0.72 m for MERIT. It follows that the product accuracy could be improved by acquiring additional LiDAR and using FABDEM for areas where LiDAR is not available.

Second, the structure of the FwDET model itself introduced uncertainty. While the model was designed to handle the large spatial and temporal domain of the study area, it may struggle to accurately represent areas distant from flood perimeters or capture surface water elevation in areas with steep banks. Incorporating additional hydraulic processes into the model, alongside more precise inputs, could enhance the level of detail and accuracy in the results.

Third, the accuracy of the flood water depth has been assessed using hydrodynamic models, which are calibrated based on observed flood extents and depths. Although these models provide detailed estimates for specific events, alternative validation methods such as comparing with streamflow gauge levels or field data could provide additional avenues for validating the models and enhancing confidence in the results.

Addressing these sources of uncertainty would strengthen the reliability and usefulness of the flood depth products. Further improvements in input data quality, model structure, and validation techniques are recommended to refine and enhance the accuracy of the FwDET model, thereby increasing its utility for understanding the role of flooding in the floodplain ecosystem in the MDB.

The methodology shows promise in estimating flooding in regions with limited or no hydrological data. It can be readily applied to ephemeral rivers in semi-arid areas with minimal adjustments. However, in regions characterised by thick cloud cover, such as monsoonal systems, alternative methods are needed to acquire flood extents. Similarly, in regions with permanent water bodies, obtaining accurate bathymetry data becomes essential for precise water depth estimation.

## Usage Notes

The two-monthly flood depth product provides a visualisation of specific events and a longitudinal perspective on flooding across the MDB. For users interested in specific events at a location, use the geospatial visualisation platform to confirm with local experts that the product has captured flooding in the areas expected to be inundated. The geospatial visualisation platform can be accessed through a web browser at https://map.csiro.easi-eo.solutions/. In the web browser, load the product by clicking ‘+Explore map data’, search the catalogue for ‘Flood Depth’ and click the ‘+’ next to the latest version of the flood depth product. The product will become visible once zoomed into a location of interest (inside the MDB). When satisfied, the maximum flood depth product for specific dates can be downloaded from CSIRO’s Data Access Portal (DAP) for ingestion into a GIS application. If confirming with local experts is not practicable, alternatives include searching archives such as Geoscience Australia’s Australian Flood Risk Information Portal, aerial photography archives – e.g. New South Wales Historical Satellite Imagery, media reports or social media.

For researchers interested in the longitudinal perspective on flooding across the MDB, it is necessary to access the whole dataset from CSIRO’s DAP. A Python Jupyter Notebook (example_water_depth.ipynb) provided as part of the code gives an example of selecting time periods (2021–2022), spatially sub-setting the data (e.g. near Macquarie Marshes Nature Reserve), visualising recent events and undertaking rudimentary statistical analysis. The notebook provides an example of calculating a short timeseries of water volumes and incorporating bias-correction.

The study’s findings have substantial broader impacts, benefiting communities, flood managers, decisionmakers, environmental conservation, and stakeholders in the MDB. The dataset supports evidence-based decision-making and ongoing research. For example, establishing the connections between climate change and floodplain inundation, where understanding climate change is an identified need of the 2026 Murray Darling Basin Plan Review.

## Data Availability

Version 1.0 of the water-depth-estimation code used for calculating FwDET is available under GPLv3 licensing at https://github.com/csiro-hydroinformatics/water-depth-estimation. The repository also contains a Jupyter notebook (notebooks/example_water_depth.ipynb), which is useful for exploring the water depth outputs.
